# Integrated Production and Distribution Scheduling Problems Related to Fixed Delivery Departure Dates and Weights of Late Orders

**DOI:** 10.1155/2015/804968

**Published:** 2015-02-16

**Authors:** Shanlin Li, Maoqin Li

**Affiliations:** Department of Mathematics, Taizhou University, Taizhou, Zhejiang 317000, China

## Abstract

We consider an integrated production and distribution scheduling problem faced by a typical make-to-order manufacturer which relies on a third-party logistics (3PL) provider for finished product delivery to customers. In the beginning of a planning horizon, the manufacturer has received a set of orders to be processed on a single production line. Completed orders are delivered to customers by a finite number of vehicles provided by the 3PL company which follows a fixed daily or weekly shipping schedule such that the vehicles have fixed departure dates which are not part of the decisions. The problem is to find a feasible schedule that minimizes one of the following objective functions when processing times and weights are oppositely ordered: (1) the total weight of late orders and (2) the number of vehicles used subject to the condition that the total weight of late orders is minimum. We show that both problems are solvable in polynomial time.

## 1. Introduction

An increasing number of companies now adopt make-to-order business models in which products are custom-made and delivered to customers within a very short lead time directly from the factory. Consequently, there is little or no finished product inventory in the supply chain such that production and outbound distribution are intimately linked and must be scheduled jointly. A majority of the companies worldwide rely on the 3PL providers for their daily distribution and other logistics needs [1]. 3PL providers often follow a fixed daily or weekly schedule for serving their customers. For example, many package delivery service providers such as UPS and FedEx have daily fixed package pickup times; and most 3PL rail, ocean, and air freight service providers have a fixed weekly schedule for a specific origin-destination pair.

In this paper, we study integrated production and outbound distribution scheduling decisions commonly faced by many manufacturers that operate in a make-to-order mode and rely on a 3PL provider for finished product delivery to customers where the 3PL provider follows a fixed delivery schedule. Examples of such manufacturers include most high-end custom-made consumer electronics product manufacturers based in Asia that rely on air flights (which have fixed departure times) to deliver finished products to the US and European markets. The production and distribution scheduling problem faced by such a manufacturer can be described as follows. At the beginning of a planning horizon, the manufacturer has received a set *J* = {*J*_1_, *J*_2_,…, *J*_*n*_} of *n* independent orders from its customers to be processed on a single assembly line. Order *J*_*i*_ has a processing time *p*_*i*_, a weight *w*_*i*_ and a desired due date *d*_*i*_ which is negotiated and agreed on by the manufacturer and the customer who placed the order. Finished orders are delivered by vehicles which have fixed departure times. In the planning horizon, there are *z* possible vehicle departure time instants *T*_1_, *T*_2_,…, *T*_*z*_, whereby at time *T*_*j*_, 1 ≤ *j* ≤ *z*, there are *v*_*j*_ vehicles available for delivery. In the air flight case, each vehicle represents an air freight container. Based on a contractual agreement between the manufacturer and the 3PL provider, the manufacturer can use a certain number (e.g., *v*_*j*_) of containers available on a given flight with departure time *T*_*j*_. Usually the 3PL provider charges the manufacturer a fixed transportation cost for each air freight container used. Thus, the total transportation cost is represented by the total number of vehicles (i.e., total number of containers) used. Each order is packaged into a standard-size pallet for delivery convenience regardless of the order size. Each vehicle can deliver at most *C* orders (e.g., in the air flight case, each container can hold up to *C* pallets). The *v*_*j*_ vehicles can only deliver orders that are completed by time *T*_*j*_. A feasible schedule is one in which each order has completed processing and delivered by one of the available vehicles. Without loss of generality, we may assume that *T*_*z*_ ≥ ∑_*i*=1_^*n*^ *p*_*i*_; otherwise, there is at least one order that cannot be delivered and hence there is no feasible schedule.

In a given feasible schedule, if order *J*_*i*_ is delivered at time *T*_*j*_ and *T*_*j*_ > *d*_*i*_, we define *U*_*i*_ to be 1; if order *J*_*i*_ is delivered at time *T*_*j*_ and *T*_*j*_ ≤ *d*_*i*_, we define *U*_*i*_ to be 0. We say in a given feasible schedule an order *J*_*i*_ is* early* if *U*_*i*_ = 0 and* late* if *U*_*i*_ = 1. The minimum total weight of late orders ∑_*i*=1_^*n*^ *w*_*i*_ *U*_*i*_ measures the delivery timeliness relative to the customers desired due dates and is one of the most commonly used measurements in practice. The problem of minimizing ∑_*i*=1_^*n*^ *w*_*i*_ *U*_*i*_ is NP-hard as it contains the NP-hard classical single-machine total weight of late orders scheduling problem [2] as a special case when the delivery part is not considered. In this paper, we consider the special case of the problem in which processing times and weights are oppositely ordered; that is, if *w*_*i*_ < *w*_*j*_, then *p*_*i*_ ≥ *p*_*j*_ for all *J*_*i*_, *J*_*j*_ ∈ *J*. That processing times and weights are oppositely ordered is the case in many practical applications. For example, when production resources are comparative shortage, one would prefer the order with shorter processing time. Our problem is to find a feasible schedule that minimizes one of the following objective functions: (1)∑_*i*=1_^*n*^ *w*_*i*_ *U*_*i*_ and (2) the number of vehicles used subject to the condition that ∑_*i*=1_^*n*^ *w*_*i*_ *U*_*i*_ is minimum. We show that all two problems are solvable in polynomial time.

Research on integrated production and outbound distribution scheduling problems is relatively recent but has attracted a rapidly growing interest in the last several years [3]. In most of the problems considered in the literature, vehicle departure times are not fixed and need to be determined along with other decisions. Only a handful of problems considered in the literature involve fixed vehicle departure times. Such problems can be classified into two types based on vehicle availability. One type assumes that there are infinite number of vehicles available at each departure time, whereas the other type assumes that there are a limited number of vehicles available at each departure time. Stecke and Zhao [4], Melo and Wolsey [5], and Zhong et al. [6] all consider similar problems with an infinite number of vehicles where each order has a deadline which has to be satisfied and the objective is to minimize the total transportation cost. Their problems differ slightly in the structure of the transportation cost. Since the focus of this paper is on problems with a finite number of vehicles, we do not review these papers in detail.

Li et al. [7–9] and Zandieh and Molla-Alizadeh-Zavardehi [10] study several similar problems with a finite number of vehicles at each departure time which are all motivated by applications involving synchronizing assembly operations of consumer electronics products such as PCs and air transportation schedules. Orders may have different sizes and the capacity of a vehicle is measured by the total size (weight or volume) of orders that it can carry. There is an earliness or tardiness penalty if an order is delivered earlier or later than the due date. The objective is to minimize the total transportation cost and total weighted earliness and tardiness penalty. Li et al. [9] consider the case where all the orders are processed on a number of parallel production lines, whereas the other papers consider the case with a single production line. The problems are strongly NP-hard as they contain the strongly NP-hard classical single-machine total weighted tardiness scheduling problem [11] as a special case when the delivery part is not considered. These papers propose various heuristics for solving their problems. Wang et al. [12] study a problem with a finite number of vehicles which involves coordinating mail processing and distribution schedules at a mail processing and distribution center. The objective is to minimize the total unused vehicle capacity. The authors show that this problem is strongly NP-hard and propose dispatching rules and heuristics.

Fu et al. [13] consider a problem where there is a limit on the total delivery capacity at each departure time. Each order has a delivery departure deadline, a production window, a size, and a profit. The problem is to select a subset of orders to accept so as to maximize the total profit of the accepted orders under the constraint that each accepted order is processed within its production window, the delivery of this order is departed by its delivery departure deadline, and the total size of the orders delivered at each departure time does not exceed the available vehicle capacity limit. The problem is strongly NP-hard as it contains the bin packing problem as its special case when only the delivery part is considered. The authors propose a polynomial time approximation scheme for the problem.

Leung and Chen [14] discuss an integrated production and distribution scheduling problem. In the beginning of a planning horizon, the manufacturer has received a set of orders to be processed on a single production line. Completed orders are delivered to customers by a finite number of vehicles provided by the 3PL company which follows a fixed daily or weekly shipping schedule such that the vehicles have fixed departure dates. The problem is to find a feasible schedule that minimizes one of the following objective functions: (a) the maximum lateness of orders, (b) the number of vehicles used subject to the condition that the maximum lateness is minimum, and (c) the weighted sum of the maximum lateness and the number of vehicles used. They show that all three problems are solvable in polynomial time.

The remainder of this paper is organized as follows. In Section 2, we give a simple algorithm to check the feasibility of a given instance of the problem. In Sections 3 and 4, we give polynomial time algorithms to solve problems (1) and (2), respectively. We conclude the paper in Section 5.

## 2. Feasibility

Given an instance of the problem, we first need to determine whether there is any feasible schedule. The following Algorithm FD come from Leung and Chen [14] and are stated. The idea is to schedule the orders in Smallest-Processing-Time first (SPT) order. Let *S* be a SPT schedule. Let *S*(*T*_*i*_, *T*_*j*_) denote the set of orders completed in the interval (*T*_*i*_, *T*_*j*_] in *S*. Let *T*_*z*+1_ be any integer greater than *T*_*z*_. We then assign the orders to the vehicles by the following algorithm.

*Algorithm FD*

*Input*. An SPT schedule *S*. For each departure time *T*_*j*_, for 1 ≤ *j* ≤ *z*, there are *v*_*j*_ vehicles available for delivery at *T*_*j*_. 

*Output*. “Yes” if it is possible to deliver all the orders in the schedule *S*, “No” otherwise. 

*Method*

1. *S*(*T*_*z*_, *T*_*z*+1_): = *∅*; *T*_0_ : = 0.
2. For *j* = 1 to *z* do the following:
  (a) assign the orders in *S*(*T*_*j*−1_, *T*_*j*_) to one of the *v*_*j*_ vehicles available at time *T*_*j*_;
   after an order is assigned to a vehicle, it is removed from *S*(*T*_*j*−1_, *T*_*j*_);
  (b) if all of the *v*_*j*_ vehicles are full and there is still at least one unassigned order in *S*(*T*_*j*−1_, *T*_*j*_), then put all the unassigned orders into *S*(*T*_*j*_, *T*_*j*+1_);
3. if *S*(*T*_*z*_, *T*_*z*+1_) = *∅* then return “Yes,” else return “No.”

If the algorithm returns “Yes,” then there is a feasible schedule; otherwise, there is no feasible schedule. The overall running time of the algorithm is *O*(*n*log⁡⁡*n*) time.

Let (*S*_1_, *S*_2_) be a SPT schedule of the instance of the problem. Clearly, there is no feasible schedule for the instance of the problem if |*S*_2_| = ∑_*j*=*k*_^*z*^ *v*_*j*_ *C* and ∑_*J*_*i*_∈*S*_1__ *p*_*i*_ > *T*_*k*−1_, where 1 ≤ *k* ≤ *z*. In the remainder of this paper, we will assume that there is a feasible schedule for the given instance.

## 3. Total Weight of Late Orders

In this section we give a polynomial-time algorithm to solve the total weight of late orders problem. For each 1 ≤ *i* ≤ *n*, let $\bar{d_{i}}=\max\{T_{m}|T_{m}\leq d_{i},1\leq m\leq z\}$ and for each 1 ≤ *j* ≤ *z*, let $N_{0,j}=\{J_{i}|\bar{d_{i}}=T_{j},1\leq i\leq n\}$. Clearly, an order *J*_*i*_ ∈ *N*_0,*j*_ is early if and only if it is completed and delivered by time *T*_*j*_ for a given schedule. The following algorithm decides whether there is a feasible schedule that minimizes the total weight of late orders. To break ties when sequencing the orders in decreasing order of their weights, we employ the last-in first rule; that is, we arrange order *J*_*j*_ before order *J*_*i*_ if *J*_*j*_ is merged into a set of orders *S* and *w*_*j*_ = *w*_*i*_, where *J*_*i*_ ∈ *S*. 

*Algorithm WF*

*Input*. *J* and *N*_0,1_,…, *N*_0,*z*_. 

*Output*. A feasible schedule that minimizes the total weight of late orders. 

*Method*

1. *P* = ∑_*i*=1_^*n*^ *p*_*i*_; *t* : = 0. *T*_0_ : = 0. *N*_0,0_ : = *∅*.
2.  For *j* = *z* down to 1 do the following.
  (2.1) Let *R*_*t*,*j*_ be all the orders in *N*_*t*,*j*_, arranged in decreasing order of their weights.
  (2.2) If *v*_*j*_ *C* < |*R*_*t*,*j*_|, then update *N*_*t*,*j*−1_ : = *N*_*t*,*j*−1_ ∪ *F*_*t*,*j*_ and *R*_*t*,*j*_ : = *R*_*t*,*j*_∖*F*_*t*,*j*_, where *F*_*t*,*j*_ are the first |*R*_*t*,*j*_| − *v*_*j*_ *C* orders from *R*_*t*,*j*_.
  (2.3) *p* : = ∑_*J*_*i*_∈*R*_*t*,*j*__ *p*_*i*_.
  (2.4) Schedule the orders in *R*_*t*,*j*_ from time *P* − *p* to *P*. These orders will be delivered by the vehicles available at time *T*_*j*_.
  (2.5) *P* : = *P* − *p*.
  (2.6) If *P* > *T*_*j*−1_, find *J*_*l*_*t*__ ∈ *N*_*t*,*j*_*l*__ such that *w*_*l*_*t*__ = min⁡{*w*_*i*_∣*J*_*i*_ ∈ ⋃_*i*=0_^*j*−1^ *N*_*t*,*i*_}, where 0 ≤ *j*_*l*_ ≤ *j* − 1. Update *N*_*t*,0_ : = *N*_*t*,0_, *N*_*t*,1_ : = *N*_*t*,1_,…, *N*_*t*,*j*_*l*_−1_ : = *N*_*t*,*j*_*l*_−1_, *N*_*t*,*j*_*l*__ : = *N*_*t*,*j*_*l*__∖{*J*_*l*_*t*__}, *N*_*t*,*j*_*l*_+1_ : = *N*_*t*,*j*_*l*_+1_,…, *N*_*t*,*j*−1_ : = *N*_*t*,*j*−1_, *N*_*t*,*j*_ : = *R*_*t*,*j*_,…, *N*_*t*,*z*−1_ : = *R*_*t*,*z*−1_, *N*_*t*,*z*_ : = *R*_*t*,*z*_ ∪ {*J*_*l*_*t*__}, and *t* : = *t* + 1; return to (2).
3. Stop. The schedule (*R*_*t*,1_,…, *R*_*t*,*z*_) is an optimal feasible schedule, where the orders in *R*_*t*,*j*_ will be delivered by the vehicles available at time *T*_*j*_ and ∑_*i*=0_^*t*−1^ *w*_*l*_*i*__ = min⁡∑*w*_*j*_ *U*_*j*_.

Algorithm WF consists of *t* + 1 iterations. For ease of presentation, we present the detailed output data obtained by the *t* + 1 iterations as follows:

(1) R0,j0,…,R0,z,Jl0,wl0;…;Rt−1,jt−1,…,Rt−1,z,Jlt−1,wlt−1;Rt,1,…,Rt,z,

where for *k* = 0,1,…, *t* − 1, 1 ≤ *j*_*k*_ ≤ *z*, *P* − ∑_*J*_*i*_∈⋃_*j*=*j*_*k*__^*z*^*R*_*k*,*j*__ *p*_*i*_ > *T*_*j*_*k*_−1_, *J*_*l*_*k*__ ∈ *J*∖⋃_*j*=*j*_*k*__^*z*^ *R*_*k*,*j*_, and *w*_*l*_*k*__ = min⁡{*w*_*i*_∣*J*_*i*_ ∈ *J*∖⋃_*j*=*j*_*k*__^*z*^ *R*_*k*,*j*_}.

The following lemma describes some properties of the data obtained by Algorithm WF.

**Lemma 1 .**

Let the data in (1) be obtained by Algorithm WF. All of the following hold:

- *j*_0_ ≥ *j*_1_ ≥ ⋯≥*j*_*t*_ = 1,
- *w*_*l*_0__ ≤ *w*_*l*_1__ ≤ ⋯≤*w*_*l*_*t*−1__,
- for each 0 ≤ *k* ≤ *t* − 1 and *k* + 1 ≤ *i* ≤ *t*, *J*_*l*_*k*__ ∈ ⋃_*j*=*j*_*k*__^*z*^ *R*_*i*,*j*_ and is a late order if the orders in *R*_*i*,*j*_ are delivered at time *T*_*j*_.

**Proof**

Let (*R*_0,*j*_0__,…, *R*_0,*z*_), *J*_*l*_0__, and *w*_*l*_0__ be the output data obtained by the first iteration of Algorithm WF. Then, we have that *P* − ∑_*J*_*i*_∈⋃_*j*=*m*_^*z*^*R*_0,*j*__ *p*_*i*_ ≤ *T*_*m*−1_, |*R*_0,*m*_| ≤ *v*_*m*_ *C* for *m* = *z*, *z* − 1,…, *j*_0_ + 1, and *P* − ∑_*J*_*i*_∈⋃_*j*=*j*_0__^*z*^*R*_0,*j*__ *p*_*i*_ > *T*_*j*_0_−1_, |*R*_0,*j*_0__| ≤ *v*_*j*_0__ *C*. We now run the second iteration on the data (*N*_1,0_, *N*_1,1_,…, *N*_1,*z*_). It follows from Steps (2.1) and (2.2) that *R*_1,*z*_ = *R*_0,*z*_ ∪ {*J*_*l*_0__} if |*R*_0,*z*_| < *v*_*z*_ *C* and *R*_1,*z*_ = *R*_0,*z*_ ∪ {*J*_*l*_0__}∖{*J*_*l*_} otherwise, where *w*_*l*_ = max⁡{*w*_*i*_∣*J*_*i*_ ∈ *R*_0,*z*_ ∪ {*J*_*l*_0__}}. Since order processing times and order weights are oppositely ordered, we have *p*_*l*_ = min⁡{*p*_*i*_∣*J*_*i*_ ∈ *R*_0,*z*_ ∪ {*J*_*l*_0__}}. In either case, ∑_*J*_*i*_∈*R*_1,*z*__ *p*_*i*_ ≥ ∑_*J*_*i*_∈*R*_0,*z*__ *p*_*i*_. This implies that *P* − ∑_*J*_*i*_∈*R*_1,*z*__ *p*_*i*_ ≤ *P* − ∑_*J*_*i*_∈*R*_0,*z*__ *p*_*i*_ ≤ *T*_*z*−1_. Further, we have that for *m* = *z* − 1,…, *j*_0_,

(2) ⋃j=mzR1,j=⋃j=mzR0,j∪Jl0 if  ∑j=mzR0,j<∑j=mzvjC,

(3) ⋃j=mzR1,j=⋃j=mzR0,j∪Jl0∖Jl if  ∑j=mzR0,j=∑j=mzvjC,

where *p*_*l*_ = min⁡{*p*_*i*_∣*J*_*i*_ ∈ ⋃_*j*=*m*_^*z*^ *R*_0,*j*_ ∪ {*J*_*l*_0__}}. This implies that for *m* = *z* − 1,…, *j*_0_ + 1, *P* − ∑_*J*_*i*_∈⋃_*j*=*m*_^*z*^*R*_1,*j*__ *p*_*i*_ ≤ *P* − ∑_*J*_*i*_∈⋃_*j*=*m*_^*z*^*R*_0,*j*__ *p*_*i*_ ≤ *T*_*m*−1_. Thus, *j*_0_ ≥ *j*_1_ holds. The proof of the following inequalities in (i) is similar to that of the first inequality. We conclude that (i) holds.

By Algorithm WF, we have that *J*_*l*_0__ ∈ *J*∖⋃_*j*=*j*_0__^*z*^ *R*_0,*j*_ and *J*_*l*_1__ ∈ *J*∖⋃_*j*=*j*_1__^*z*^ *R*_1,*j*_, where *w*_*l*_0__ = min⁡{*w*_*i*_∣*J*_*i*_ ∈ *J*∖⋃_*j*=*j*_0__^*z*^ *R*_0,*j*_} and *w*_*l*_1__ = min⁡{*w*_*i*_∣*J*_*i*_ ∈ *J*∖⋃_*j*=*j*_1__^*z*^ *R*_1,*j*_}. Due to (i), *j*_0_ ≥ *j*_1_. By (2), ⋃_*j*=*j*_0__^*z*^ *R*_1,*j*_ = ⋃_*j*=*j*_0__^*z*^ *R*_0,*j*_ ∪ {*J*_*l*_0__} if ∑_*j*=*j*_0__^*z*^|*R*_0,*j*_| < ∑_*j*=*j*_0__^*z*^ *v*_*j*_ *C*. In the case, since *J*∖⋃_*j*=*j*_0__^*z*^ *R*_0,*j*_⊇*J*∖⋃_*j*=*j*_1__^*z*^ *R*_1,*j*_, we have that *w*_*l*_0__ ≤ *w*_*l*_1__. By (3), ⋃_*j*=*j*_0__^*z*^ *R*_1,*j*_ = ⋃_*j*=*j*_0__^*z*^ *R*_0,*j*_ ∪ {*J*_*l*_0__}∖{*J*_*l*_} if ∑_*j*=*j*_0__^*z*^|*R*_0,*j*_| = ∑_*j*=*j*_0__^*z*^ *v*_*j*_ *C*, where *w*_*l*_ = max⁡{*w*_*i*_∣*J*_*i*_ ∈ ⋃_*j*=*j*_0__^*z*^ *R*_0,*j*_ ∪ {*J*_*l*_0__}}. In the case, since *J*∖⋃_*j*=*j*_0__^*z*^ *R*_0,*j*_⊇{*J*_*l*_} ∪ *J*∖⋃_*j*=*j*_1__^*z*^ *R*_1,*j*_, along with *w*_*l*_ ≥ *w*_*l*_0__, we have that *w*_*l*_0__ ≤ *w*_*l*_1__. Thus, the first inequality *w*_*l*_0__ ≤ *w*_*l*_1__ in (ii) holds. The proof of the following inequalities in (ii) is similar to that of the first inequality. We conclude that (ii) holds.

For any *k* ∈ {0,1,…, *t* − 1}, suppose *J*_*l*_*k*__ ∈ *N*_0,*k*_0__. Clearly, *J*_*l*_*k*__ is a late order if it is delivered at time *T*_*j*_, where *k*_0_ < *j* ≤ *z*. To show (iii), there are two cases to consider: (a) *k*_0_ < *j*_*k*_ and (b) *k*_0_ ≥ *j*_*k*_.

(a) Consider *k*_0_ < *j*_*k*_. Assume that *J*_*l*_*k*__ ∈ ⋃_*j*=*j*_*k*__^*z*^ *R*_*k*+1,*j*_ does not hold. It follows from the argument of (i) that ∑_*j*=*j*_*k*__^*z*^|*R*_*k*,*j*_| = ∑_*j*=*j*_*k*__^*z*^ *v*_*j*_ *C*, ⋃_*j*=*j*_*k*__^*z*^ *R*_*k*+1,*j*_ = ⋃_*j*=*j*_*k*__^*z*^ *R*_*k*,*j*_, and *w*_*l*_*k*__ = max⁡{*w*_*i*_∣*J*_*i*_ ∈ ⋃_*j*=*j*_*k*__^*z*^ *R*_*k*,*j*_ ∪ {*J*_*l*_*k*__}}; that is, *p*_*l*_*k*__ = min⁡{*p*_*i*_∣*J*_*i*_ ∈ ⋃_*j*=*j*_*k*__^*z*^ *R*_*k*,*j*_ ∪ {*J*_*l*_*k*__}}. This, along with *w*_*l*_*k*__ = min⁡{*w*_*i*_∣*J*_*i*_ ∈ *J*∖⋃_*j*=*j*_*k*__^*z*^ *R*_*k*,*j*_}, that is, *p*_*l*_*k*__ = max⁡{*p*_*i*_∣*J*_*i*_ ∈ *J*∖⋃_*j*=*j*_*k*__^*z*^ *R*_*k*,*j*_}, implies that, for any *J*_*i*_1__ ∈ *J*∖⋃_*j*=*j*_*k*__^*z*^ *R*_*k*,*j*_ and *J*_*i*_2__ ∈ ⋃_*j*=*j*_*k*__^*z*^ *R*_*k*,*j*_, *p*_*i*_1__ ≤ *p*_*i*_2__. Thus, we have that there is a SPT schedule (*S*_1_, *S*_2_) such that |*S*_2_| = ∑_*j*=*j*_*k*__^*z*^ *v*_*j*_ *C* and ∑_*J*_*i*_∈*S*_1__ *p*_*i*_ > *T*_*j*_*k*_−1_, where *S*_1_ = *J*∖⋃_*j*=*j*_*k*__^*z*^ *R*_*k*,*j*_ and *S*_2_ = ⋃_*j*=*j*_*k*__^*z*^ *R*_*k*,*j*_. This implies that there is no feasible schedule for the instance of the problem, which contradicts the assumption that there is a feasible schedule for the given instance. Thus, *J*_*l*_*k*__ ∈ ⋃_*j*=*j*_*k*__^*z*^ *R*_*k*+1,*j*_ and is a late order if the orders in *R*_*k*+1,*j*_ are delivered at time *T*_*j*_. Further, it follows from the argument of (i) that ⋃_*j*=*j*_*k*__^*z*^ *R*_*k*+2,*j*_ = ⋃_*j*=*j*_*k*__^*z*^ *R*_*k*+1,*j*_ ∪ {*J*_*l*_*k*+1__} if ∑_*j*=*j*_*k*__^*z*^|*R*_*k*+1,*j*_| < ∑_*j*=*j*_*k*__^*z*^ *v*_*j*_ *C* and  ⋃_*j*=*j*_*k*__^*z*^ *R*_*k*+2,*j*_ = ⋃_*j*=*j*_*k*__^*z*^ *R*_*k*+1,*j*_ ∪ {*J*_*l*_*k*+1__}∖{*J*_*l*_} if ∑_*j*=*j*_*k*__^*z*^|*R*_*k*+1,*j*_| = ∑_*j*=*j*_*k*__^*z*^ *v*_*j*_ *C*, where *w*_*l*_ = max⁡{*w*_*i*_∣*J*_*i*_ ∈ ⋃_*j*=*j*_*k*__^*z*^ *R*_*k*+1,*j*_ ∪ {*J*_*l*_*k*+1__}}. This, along with *w*_*l*_*k*__ ≤ *w*_*l*_*k*+1__ and *J*_*l*_*k*__ merged into ⋃_*j*=*j*_*k*__^*z*^ *R*_*k*+1,*j*_ before *J*_*l*_*k*+1__, implies that *J*_*l*_*k*__ ∈ ⋃_*j*=*j*_*k*__^*z*^ *R*_*k*+2,*j*_ and is a late order if the orders in *R*_*k*+2,*j*_ are delivered at time *T*_*j*_. Similarly, we can show *J*_*l*_*k*__ ∈ ⋃_*j*=*j*_*k*__^*z*^ *R*_*i*,*j*_ and is a late order if the orders in *R*_*i*,*j*_ are delivered at time *T*_*j*_ for *i* = *k* + 3,…, *t*.

(b) Consider *k*_0_ ≥ *j*_*k*_. Note the fact that order *J*_*l*_*k*__ is pushed by the iterations from *N*_0,*k*_0__ into *J*∖⋃_*j*=*j*_*k*__^*z*^ *R*_*k*,*j*_. By Steps (2.1) and (2.2), we have that |*R*_*k*,*j*_| = *v*_*j*_ *C* for *j* = *j*_*k*_,…, *k*_0_ and *w*_*l*_*k*__ = max⁡{*w*_*i*_∣*J*_*i*_ ∈ ⋃_*j*=*j*_*k*__^*k*_0_^ *R*_*k*,*j*_}. This implies *J*_*l*_*k*__ ∈ ⋃_*j*=*k*_0_+1_^*z*^ *R*_*k*+1,*j*_  (⊆⋃_*j*=*j*_*k*__^*z*^ *R*_*k*+1,*j*_). Otherwise, it follows from the argument of (i) that ∑_*j*=*k*_0_+1_^*z*^|*R*_*k*,*j*_| = ∑_*j*=*k*_0_+1_^*z*^ *v*_*j*_ *C*, ⋃_*j*=*k*_0_+1_^*z*^ *R*_*k*+1,*j*_ = ⋃_*j*=*k*_0_+1_^*z*^ *R*_*k*,*j*_, and *w*_*l*_*k*__ = max⁡{*w*_*i*_∣*J*_*i*_ ∈ ⋃_*j*=*k*_0_+1_^*z*^ *R*_*k*,*j*_ ∪ {*J*_*l*_*k*__}}, and then we have that ∑_*j*=*j*_*k*__^*z*^|*R*_*k*,*j*_| = ∑_*j*=*j*_*k*__^*z*^ *v*_*j*_ *C*, ⋃_*j*=*j*_*k*__^*z*^ *R*_*k*+1,*j*_ = ⋃_*j*=*j*_*k*__^*z*^ *R*_*k*,*j*_, and *w*_*l*_*k*__ = max⁡{*w*_*i*_∣*J*_*i*_ ∈ ⋃_*j*=*j*_*k*__^*z*^ *R*_*k*,*j*_ ∪ {*J*_*l*_*k*__}}. Similar to the argument of case (a), we can derive a contradiction. Similarly, we can show *J*_*l*_*k*__ ∈ ⋃_*j*=*j*_*k*__^*z*^ *R*_*i*,*j*_ and is a late order if the orders in *R*_*i*,*j*_ are delivered at time *T*_*j*_ for *i* = *k* + 2,…, *t*. This ends the proof for (iii).

**Theorem 2 .**

Algorithm WF correctly finds a feasible schedule that minimizes the total weight of late orders in *O*(*zn* ^2^log⁡⁡*n*) time.

**Proof**

We first prove that ⋃_*j*=*j*_*k*__^*z*^ *R*_*k*,*j*_ has not only exactly *k* late orders {*J*_*l*_0__,…, *J*_*l*_*k*−1__} but also the maximum total processing time and the minimum total weight ∑_*i*=0_^*k*−1^ *w*_*l*_*i*__ of late orders among all schedules for *k* = 0,1,…, *t* − 1, where {*J*_*l*_0__,…, *J*_*l*_*k*−1__} = *∅* and ∑_*i*=0_^*k*−1^ *w*_*l*_*i*__ = 0 if *k* = 0. By the definition of *N*_0,*z*_ and Steps (2.1) and (2.2) of Algorithm WF, we have that *R*_0,*z*_ is a set of early orders delivered at time *T*_*z*_ with the maximum total processing time among all schedules. Similarly, we have that ⋃_*j*=*m*_^*z*^ *R*_0,*j*_ is a set of early orders with the maximum total processing time among all schedules for *m* = *z* − 1,…, *j*_0_.

By (2) and (3), we have that ⋃_*j*=*j*_0__^*z*^ *R*_1,*j*_ = ⋃_*j*=*j*_0__^*z*^ *R*_0,*j*_ ∪ {*J*_*l*_0__} if ∑_*j*=*j*_0__^*z*^|*R*_0,*j*_| < ∑_*j*=*j*_0__^*z*^ *v*_*j*_ *C* and ⋃_*j*=*j*_0__^*z*^ *R*_1,*j*_ = ⋃_*j*=*j*_0__^*z*^ *R*_0,*j*_ ∪ {*J*_*l*_0__}∖{*J*_*l*_} if ∑_*j*=*j*_0__^*z*^|*R*_0,*j*_| = ∑_*j*=*j*_0__^*z*^ *v*_*j*_ *C* where *w*_*l*_ = max⁡{*w*_*i*_∣*J*_*i*_ ∈ ⋃_*j*=*j*_0__^*z*^ *R*_0,*j*_ ∪ {*J*_*l*_0__}}. This, along with (iii) of Lemma 1, *w*_*l*_0__ = min⁡{*w*_*i*_∣*J*_*i*_ ∈ *J*∖⋃_*j*=*j*_0__^*z*^ *R*_0,*j*_} and ⋃_*j*=*j*_0__^*z*^ *R*_0,*j*_ being a set of early orders with the maximum total processing time among all schedules, implies that ⋃_*j*=*j*_0__^*z*^ *R*_1,*j*_ has not only exactly a late order *J*_*l*_0__ but also the maximum total processing time and the minimum weight *w*_*l*_0__ among all schedules. Further, by Steps (2.1) and (2.2) of Algorithm WF, we have that ⋃_*j*=*m*_^*z*^ *R*_1,*j*_ has not only exactly a late order *J*_*l*_0__ but also the maximum total processing time and the minimum weight *w*_*l*_0__ among all schedules for *m* = *j*_0_ − 1,…, *j*_1_. Similarly, we can show the result for *k* = 2,…, *t* − 1.

We below prove that (*R*_*t*,1_,…, *R*_*t*,*z*_) is a feasible schedule minimizing the total weight of late orders. By Algorithm WF, the feasibility is obvious. For any feasible schedule *S* = (*R*_1_,…, *R*_*z*_), where the orders in *R*_*j*_ are delivered at time *T*_*j*_ for *j* = 1,…, *z*, we have that ⋃_*j*=*j*_0__^*z*^ *R*_*j*_ contains at least a late order. Otherwise, by ∑_*J*_*i*_∈⋃_*j*=*j*_0__^*z*^*R*_*j*__ *p*_*i*_ ≤ ∑_*J*_*i*_∈⋃_*j*=*j*_0__^*z*^*R*_0,*j*__ *p*_*i*_, we have that ∑_*J*_*i*_∈*S*∖⋃_*j*=*j*_0__^*z*^*R*_*j*__ *p*_*i*_ ≥ *P* − ∑_*J*_*i*_∈⋃_*j*=*j*_0__^*z*^*R*_0,*j*__ *p*_*i*_ > *T*_*j*_0_−1_. This contradicts the feasibility of *S*. Given that ⋃_*j*=*j*_0__^*z*^ *R*_*j*_ contains at least a late order, ⋃_*j*=*j*_1__^*z*^ *R*_*j*_ contains at least two late orders. Otherwise, ⋃_*j*=*j*_1__^*z*^ *R*_*j*_ contains a late order. By ∑_*J*_*i*_∈⋃_*j*=*j*_1__^*z*^*R*_*j*__ *p*_*i*_ ≤ ∑_*J*_*i*_∈⋃_*j*=*j*_1__^*z*^*R*_1,*j*__ *p*_*i*_, we have that ∑_*J*_*i*_∈*S*∖⋃_*j*=*j*_1__^*z*^*R*_*j*__ *p*_*i*_ ≥ *P* − ∑_*J*_*i*_∈⋃_*j*=*j*_1__^*z*^*R*_1,*j*__ *p*_*i*_ > *T*_*j*_1_−1_. This contradicts the feasibility of *S*. Similarly, we can show that ⋃_*j*=*j*_*k*__^*z*^ *R*_*j*_ contains *k* + 1 late orders at least for *k* = 2,…, *t* − 1. Thus, (*R*_*t*,1_,…, *R*_*t*,*z*_) is a feasible schedule minimizing the number of late orders. This, along with ∑_*i*=0_^*t*−1^ *w*_*l*_*i*__ being the minimum total weight, implies that Algorithm WF correctly finds a feasible schedule that minimizes the total weight of late orders.

We now show that the algorithm can be implemented to run in *O*(*zn* ^2^log⁡⁡*n*) time. The algorithm consists of *n* + 1 iterations at most since there are *n* late orders at most. To calculate the complexity of the algorithm we first calculate in a preprocessing step the sets $\left(\bar{d_{1}},\ldots ,\bar{d_{n}}\right)$ and (*N*_0,1_,…, *N*_0,*z*_). The calculation of the set $\left(\bar{d_{1}},\ldots ,\bar{d_{n}}\right)$ takes *O*(*n*log⁡⁡*z*). The calculation of the set (*N*_0,1_,…, *N*_0,*z*_) takes *O*(*n*log⁡⁡*z*) also. Step (1) takes *O*(*n*) time. Step (2) is iterated *z* times. Within each iteration, the most time-consuming step is Step (2.1), sorting the orders in decreasing order of their weights, which takes *O*(*n*log⁡⁡*n*) time. Hence Step (2) takes *O*(*zn*log⁡⁡*n*) time. Thus, the overall running time of the algorithm is *O*(*zn* ^2^log⁡⁡*n*) time.

## 4. Number of Vehicles Used

In this section we show that the problem of minimizing the number of vehicles used subject to the constraint that the total weight of late orders is minimum can be solved in polynomial time. We assume that we have found the schedule that minimizes the total weight of late orders using the algorithm given in the previous section. The schedule (*R*_*t*,1_,…, *R*_*t*,*z*_) was obtained by Algorithm WF. By the fact that if |*R*_*t*,*j*_| < *v*_*j*_ *C*, then each order in ⋃_*i*=1_^*j*−1^ *R*_*t*,*i*_ is an early order and will be a late order if we push the order to be delivered by vehicles at *T*_*j*_. Thus, either there is no feasible schedule or the total weight of late orders will be increased if we push some order in *R*_*t*,*i*_ to be delivered by vehicles at *T*_*i*+1_,…, *T*_*z*_. By the optimality of ∑_*i*=0_^*t*−1^ *w*_*l*_*i*__, we have that the total weight of late orders will not be decreased if we push some order in *R*_*t*,*i*_ to be delivered by vehicles at *T*_1_,…, *T*_*i*−1_. The following algorithm finds a solution with a minimum number of vehicles used under the constraint that the total weight of late orders is minimum by pushing some orders to an earlier departure time for delivery.

*Algorithm MV*

*Input*. *z* sets of orders *R*_*t*,1_,…, *R*_*t*,*z*_ given by Algorithm WF. 

*Output*. A vehicle assignment: *V*_1_,…, *V*_*z*_, where the orders in *V*_*j*_ will be delivered by the vehicles available at time *T*_*j*_, so that the number of vehicles used is minimum. 

*Method*

1. *T*_0_ : = 0. *v*_0_ : = 0.
2. For *j* = *z* down to 1 do the following.
  - Let *V*_*j*_′ be all the orders in *R*_*t*,*j*_, arranged in nondecreasing order of their processing times, and |*V*_*j*_′| = *kC* + *r*, where *k* and *r* are nonnegative integers, and 0 ≤ *r* < *C* and *F* is the set of the first *r* orders from *V*_*j*_′.
  - If ∑_*J*_*i*_∈∪_*m*=1_^*j*−1^*R*_*t*,*m*_∪*F*_ *p*_*i*_ > *T*_*j*−1_, then update *A* : = *∅* and *V*_*j*_ : = *V*_*j*_′, and the orders in *V*_*j*_ will be delivered by the vehicles at time *T*_*j*_ and proceed to the next *j*.
  - Let *A*′ be the first *k*_0_ *C* + *r* orders from *V*_*j*_′, where *k*_0_ is the maximal nonnegative integer such that ∑_*J*_*i*_∈∪_*m*=1_^*j*−1^*R*_*t*,*m*_∪*A*′_ *p*_*i*_ ≤ *T*_*j*−1_.
  - If ⌈|*R*_*t*,*j*−1_ ∪ *A*′|/*C*⌉ ≤ *v*_*j*−1_ *C*, then update *A* : = *A*′, *R*_*t*,*j*−1_ : = *R*_*t*,*j*−1_ ∪ *A*, and *V*_*j*_ : = *V*_*j*_′∖*A* and the orders in *V*_*j*_ will be delivered by the vehicles available at time *T*_*j*_ and proceed to the next *j*.
  - Call Subalgorithm CA to computer *A*. Then update *R*_*t*,*j*−1_ : = *R*_*t*,*j*−1_ ∪ *A* and *V*_*j*_ : = *V*_*j*_′∖*A*, and the orders in *V*_*j*_ will be delivered by the vehicles at time *T*_*j*_ and proceed to the next *j*.

*Subalgorithm CA*

1. *A*′ : = the first *k*_0_ *C* + *r* orders from *V*_*j*_, arranged in nondecreasing order of their processing times.
2. Sort the orders in *R*_*t*,*j*−1_ ∪ *A*′ in ascending order of their processing times.
3. If |*R*_*t*,*j*−1_ ∪ *A*′| ≤ *v*_*j*−1_ *C*, then stop and output *A* = *A*′.
4. For *h* = 1 to *j* − 2, let *R*_*t*,*h*_′ : = *R*_*t*,*h*_.
5. *B* : = the first |*R*_*t*,*j*−1_ ∪ *A*′| − *v*_*j*−1_ *C* orders from *R*_*t*,*j*−1_ ∪ *A*′.
6. For *h* = *j* − 2 down to 1 do the following.
  (a) Sort the orders in *R*_*t*,*h*_′ ∪ *B* in ascending order of their processing times.
  (b) If ∑_*J*_*i*_∈∪_*m*=1_^*h*^*R*_*t*,*m*_′∪*B*_ *p*_*i*_ > *T*_*h*_,
    (b1) if |*A*′| < *C*, stop and output *A* = *∅*;
    (b2) update *A*′ : = the first |*A*′| − *C* orders from *A*′ and return (2).
  (c) If |*R*_*t*,*h*_′ ∪ *B*| ≤ *v*_*h*_ *C*, then stop and output *A* = *A*′.
  (d) If *h* = 1, stop and output *A* = *∅* if |*A*′| < *C* and update *A*′ : = the first |*A*′| − *C* orders from *A*′ and return (2) else.
  (e) Update *B* : = the first |*R*_*t*,*h*_′ ∪ *B*| − *v*_*h*_ *C* orders from *R*_*t*,*h*_′ ∪ *B* and proceed to the next *h*.

**Theorem 3 .**

Algorithm MV finds an optimal solution with the minimum number of vehicles used under the constraint that the total weight of late orders is minimum in *O*(*z* ^2^ *n* ^2^log⁡⁡*n*) time.

**Proof**

We first point out that the solution by Algorithm MV does not change the optimality of the total weight of late orders, since the solution is found by pushing some orders in *R*_*t*,2_,…, *R*_*t*,*z*_ to an earlier departure time for delivery.

Let *V*_*z*_ and *A* be the output data obtained by the first iteration of Algorithm MV, where *V*_*z*_ = *V*_*z*_′∖*A* and *A* is the set of the first |*A*| orders from *V*_*z*_′ (the orders of *V*_*z*_′ = *R*_*t*,*z*_ have been arranged in nondecreasing order of their processing times). Step (2)(b) corresponds to the case where ∑_*J*_*i*_∈∪_*m*=1_^*z*−1^*R*_*t*,*m*_∪*F*_ *p*_*i*_ > *T*_*z*−1_, where *F* the set of the first |*F*| orders from *V*_*z*_′ and 0 < |*F*| < *C*. In the case, It is impossible to deliver all orders in *F* by the vehicles at *T*_1_,…, *T*_*z*−1_. This, along with *F* consisting of the first |*F*| orders from *V*_*z*_′, means that the number of vehicles used at *T*_*z*_ cannot decrease by one. Thus, ⌈|*V*_*z*_′|/*C*⌉ is the minimal number of vehicles used at *T*_*z*_. However, we cannot push part of orders in *F* to be delivered by vehicles at *T*_*z*−1_,…, *T*_1_, since, if we do that, it not only does not decrease the number of vehicles used at *T*_*z*_ but also increases the amount of orders delivered by vehicles at *T*_*z*−1_,…, *T*_1_. In the case, the data *V*_*z*_ = *V*_*z*_′ and *A* = *∅* are optimal. Under the case of ∑_*J*_*i*_∈∪_*m*=1_^*z*−1^*R*_*t*,*m*_∪*F*_ *p*_*i*_ ≤ *T*_*z*−1_, Step (2)(c) computes the set of orders *A*′ which made ⌈|*V*_*z*_′|/*C*⌉ − ⌈|*A*′|/*C*⌉ the minimal number possibly of vehicles used at *T*_*z*_, where *A*′ are the first *k*_0_ *C* + *r* orders from *V*_*z*_′. This is because *k*_0_ is the maximal nonnegative integer such that ∑_*J*_*i*_∈∪_*m*=1_^*z*−1^*R*_*t*,*m*_∪*A*′_ *p*_*i*_ ≤ *T*_*z*−1_ and *A*′ consists of some small orders in *V*_*z*_′. Step (2)(d) corresponds to the case where ⌈|*R*_*t*,*z*−1_ ∪ *A*′|/*C*⌉ ≤ *v*_*z*−1_ *C*. This means that we can push all of orders in *A*′ to be delivered by vehicles at *T*_*z*−1_. Thus, in the case, the output data *A* = *A*′ and *V*_*z*_ = *V*_*z*_′∖*A* make ⌈|*V*_*z*_|/*C*⌉ the minimal number of vehicles used at *T*_*z*_. Under the case of ⌈|*R*_*t*,*z*−1_ ∪ *A*′|/*C*⌉ > *v*_*z*−1_ *C*, Step (2)(e) calls Subalgorithm CA to decide a set of orders *A* such that ⌈|*V*_*z*_|/*C*⌉ is the minimal number of vehicles used at *T*_*z*_, where *V*_*z*_ = *V*_*z*_′∖*A*. We show below that Subalgorithm CA can really do that.

Subalgorithm CA consists of *k*_0_ + 1 main iterations for the first *k*_0_ *C* + *r* orders, the first (*k*_0_ − 1)*C* + *r* orders,…, and the first *r* orders from *V*_*z*_′, respectively. We now run the first main iteration for the first *k*_0_ *C* + *r* orders *A*′ from *V*_*z*_′. Due to the corresponding case by Step (2)(e), we have that *A*′ ≠ *∅* and |*R*_*t*,*z*−1_ ∪ *A*′| > *v*_*z*−1_ *C*. We need to proceed through Step (4). Step (4) assigns the orders in *R*_*t*,*h*_ to a temporary set *R*_*t*,*h*_′ for each *h* from 1 to *j* − 2. This is necessary since Subalgorithm CA operates on *R*_*t*,*h*_′ without changing the content of *R*_*t*,*h*_. Step (5) assigns the first |*R*_*t*,*z*−1_ ∪ *A*′| − *v*_*z*−1_ *C* orders from *R*_*t*,*z*−1_ ∪ *A*′ to a set *B*. If we want to push the orders in *A*′ to be delivered by vehicles at *T*_*z*−1_,…, *T*_1_, all orders of *B* are the minimal increment undertaken by vehicles at *T*_*z*−2_,…, *T*_1_ to deliver. Now we proceed through Step (6).

Step (6) consists of *z* − 2 secondary iterations. We now run the first secondary iteration. If ∑_*J*_*i*_∈∪_*m*=1_^*z*−2^*R*_*t*,*m*_′∪*B*_ *p*_*i*_ > *T*_*z*−2_, it is impossible to deliver all orders in *B* by the vehicles at *T*_1_,…, *T*_*z*−2_. This means that the number of vehicles used at *T*_*z*_ should to be at least ⌈|*V*_*z*_′|/*C*⌉ − ⌈|*A*′|/*C*⌉ + 1. Step (6)(b1) corresponds to the case where |*A*′| < *C* and outputs *A* = *∅*. This, along with *A*′ ≠ *∅* and *V*_*z*_ = *V*_*z*_′, implies that ⌈|*V*_*z*_|/*C*⌉ is the minimal number of vehicles used at *T*_*z*_. Step (6)(b2) corresponds to the case where |*A*′| ≥ *C* and starts the second main iteration for the first (*k*_0_ − 1)*C* + *r* orders *A*′ from *V*_*z*_′ to check whether it is possible to push the orders in *A*′ to be delivered by vehicles at *T*_*z*−1_,…, *T*_1_. On the other hand, ∑_*J*_*i*_∈∪_*m*=1_^*z*−2^*R*_*t*,*m*_′∪*B*_ *p*_*i*_ ≤ *T*_*z*−2_. Step (6)(c) corresponds to the case where |*R*_*t*,*z*−2_′ ∪ *B*| ≤ *v*_*z*−2_ *C*. This means that we can push all of orders in *A*′ to be delivered by vehicles at *T*_*z*−1_ and *T*_*z*−2_. Thus, the output data *A* = *A*′ by Step (6)(c) and *V*_*z*_ = *V*_*z*_′∖*A* make ⌈|*V*_*z*_|/*C*⌉ the minimal number of vehicles used at *T*_*z*_. Otherwise, Step (6)(e) starts the second secondary iteration to check whether it is possible to push the orders in a set of the first |*R*_*t*,*z*−2_′ ∪ *B*| − *v*_*z*−2_ *C* orders from *R*_*t*,*z*−2_′ ∪ *B* to be delivered by vehicles at *T*_*z*−3_,…, *T*_1_. Similarly, we can show the result for the following secondary iterations. If needed, we proceed through the last secondary iteration. Suppose that *B* is a set of orders output by the (*z* − 3)th secondary iteration. In the case of ∑_*J*_*i*_∈*R*_*t*,1_′∪*B*_ *p*_*i*_ > *T*_1_, Step (6)(b1) outputs *A* = *∅* if |*A*′| < *C* and Step (6)(b2) starts the second main iteration for the first (*k*_0_ − 1)*C* + *r* orders *A*′ from *V*_*z*_′ else. In the case of ∑_*J*_*i*_∈*R*_*t*,1_′∪*B*_ *p*_*i*_ ≤ *T*_1_, Step (6)(c) outputs data *A* = *A*′ if |*R*_*t*,1_′ ∪ *B*| ≤ *v*_1_ *C*. Otherwise, Step (6)(d) outputs *A* = *∅* if |*A*′| < *C* and starts the second main iteration for the first (*k*_0_ − 1)*C* + *r* orders *A*′ from *V*_*z*_′ else, since it is impossible to deliver all orders in the set of the first |*R*_*t*,1_′ ∪ *B*| − *v*_1_ *C* orders from *R*_*t*,1_′ ∪ *B* by the vehicles at *T*_0_ = 0. Similarly, we can show the result for the following main iterations in Subalgorithm CA, which can really decide a set orders *A* such that ⌈|*V*_*z*_|/*C*⌉ is the minimal number of vehicles used at *T*_*z*_, where *V*_*z*_ = *V*_*z*_′∖*A*. For the following iterations in Algorithm MV, we can show the results similarly. At last, the algorithm must be able to find an optimal solution with the minimum number of vehicles used.

We now look at the time complexity of Algorithm MV. In the algorithm, Step (1) takes constant time. Step (2) is iterated *z* times. Inside the iteration loop, the most time-consuming steps are (2)(a) and (2)(e). Step (2)(a) calls for sorting the orders which takes *O*(*n*log⁡⁡*n*) time. Step (2)(e) calls Subalgorithm CA which takes *O*(*zn* ^2^log⁡⁡*n*) time. Thus, the overall time complexity of Algorithm MV is *O*(*z* ^2^ *n* ^2^log⁡⁡*n*).

## 5. Conclusion

In this paper, we have given polynomial-time algorithms for minimizing: (1) the total weight of late orders and (2) the number of vehicles used subject to the condition that the total weight of late orders is minimum. An interesting open question is whether the problem related to release dates is NP-hard or not.

## References

[1] Langley C. J., van Dort E., Sykes S. R. (2006). *2005 Third-Party Logistics: Results and Findings of the 10th Annual Study*.

[2] Karp R. M., Miller R. E., Thatcher J. W. (1972). Reducibility among combinatorial problems. *Complexity of Computer Computations*.

[3] Chen Z.-L. (2010). Integrated production and outbound distribution scheduling: review and extensions. *Operations Research*.

[4] Stecke K. E., Zhao X. (2007). Production and transportation integration for a make-to-order manufacturing company with a commit-to-delivery business mode. *Manufacturing and Service Operations Management*.

[5] Melo R. A., Wolsey L. A. (2010). Optimizing production and transportation in a commit-to-delivery business mode. *European Journal of Operational Research*.

[6] Zhong W., Chen Z.-L., Chen M. (2010). Integrated production and distribution scheduling with committed delivery dates. *Operations Research Letters*.

[7] Li K. P., Ganesan V. K., Sivakumar A. I. (2005). Synchronized scheduling of assembly and multi-destination air-transportation in a consumer electronics supply chain. *International Journal of Production Research*.

[8] Li K. P., Ganesan V. K., Sivakumar A. I. (2006). Scheduling of single stage assembly with air transportation in a consumer electronic supply chain. *Computers & Industrial Engineering*.

[9] Li K. O., Sivakumar A. I., Ganesan V. K. (2008). Complexities and algorithms for synchronized scheduling of parallel machine assembly and air transportation in consumer electronics supply chain. *European Journal of Operational Research*.

[10] Zandieh M., Molla-Alizadeh-Zavardehi S. (2009). Synchronizing production and air transportation scheduling using mathematical programming models. *Journal of Computational and Applied Mathematics*.

[11] Lenstra J. K., Rinnooy Kan A. H., Brucker P. (1977). Complexity of machine scheduling problems. *Annals of Discrete Mathematics*.

[12] Wang Q., Batta R., Szczerba R. J. (2005). Sequencing the processing of incoming mail to match an outbound truck delivery schedule. *Computers & Operations Research*.

[13] Fu B., Huo Y., Zhao H. (2012). Coordinated scheduling of production and delivery with production window and delivery capacity constraints. *Theoretical Computer Science*.

[14] Leung J. Y., Chen Z.-L. (2013). Integrated production and distribution with fixed delivery departure dates. *Operations Research Letters*.

